# Anti-drug antibodies and rheumatoid factor level in patients with rheumatoid arthritis using the infliximab biosimilar CT-P13

**DOI:** 10.1186/s41927-022-00304-9

**Published:** 2022-12-07

**Authors:** Hideo Sakane, Koichi Okamura, Makoto Inoue, Hiroshi Inoue, Yukio Yonemoto, Hirofumi Mitomi, Kosei Tsuchida, Takahito Suto, Tetsuya Kaneko, Hirotaka Chikuda

**Affiliations:** 1 Department of Orthopaedic Surgery, Gunma University Graduate School of Medicine, 3-39-22, Showamachi, Maebashi, Gunma 371-8511 Japan; 2 Department of Rheumatology, Inoue Hospital, 55, Torimachi, Takasaki, Gunma 370-0053 Japan; 3 Department of Orthopaedic Surgery, Fukaya Red Cross Hospital, 5-8-1, Kamishibacho-Nishi, Fukaya, Saitama 366-0052 Japan

**Keywords:** Anti-drug antibody, Biosimilar, CT-P13, Infliximab, Rheumatoid factor

## Abstract

**Background:**

This study evaluated the existence of anti-drug antibodies (ADAs) before and 52 weeks after switching from intravenous infliximab (IFX) to intravenous CT-P13 in patients with rheumatoid arthritis (RA).

**Methods:**

We performed a prospective observational study. Twenty-eight patients (7 males and 21 females) received intravenous CT-P13 after intravenous IFX, and the clinical data were collected from medical records. Rheumatoid factor (RF) and anti-CCP antibody were examined at baseline. At baseline and 52 weeks after the start of CT-P13 treatment, the Disease Activity Score based on the 28-joint count and the levels of C-reactive protein, matrix metalloproteinase-3, and ADA, as well as the erythrocyte sedimentation rate were evaluated. ADAs were measured using an enzyme-linked immunosorbent assay kit.

**Results:**

Seven (25%) and 6 (21.4%) cases were positive for ADAs at baseline and 52 weeks after, respectively. One case became newly positive for ADAs at week 52. Two of the ADA-positive cases became ADA-negative 52 weeks after. The ADA-positive group showed significantly higher RF values at baseline than the ADA-negative group (*p* = 0.03). No difference was observed between the ADA-positive group and the ADA-negative group regarding other clinical parameters.

**Conclusions:**

The positive rate of ADAs did not increase after switching from intravenous IFX to intravenous CT-P13. Among the patients with ADAs, a high level of RF was observed at baseline.

## Introduction

CT-P13 is a biosimilar (BS) of infliximab (IFX) approved for rheumatoid arthritis (RA). Previous clinical trials revealed that switching from IFX to a BS had no detrimental effect on disease activity compared to the IFX-continued groups [1–4]. Because the price of biosimilar is generally less than that of originator, switching from IFX to CT-P13 are strongly encouraged for economic reasons [5, 6].

The emergence of anti-drug antibodies (ADAs) with neutralizing activities has been a concern when using CT-P13 as well as IFX originator drug [1–3, 6, 7]. The prevalence of ADAs in patients receiving IFX therapy has been reported to range from 15 to 54% [3, 6, 8, 9]. Patients with ADAs for IFX had a significantly lower drug concentration than those without ADA, and the serum drug concentration was negatively correlated with the C-reactive protein (CRP) level and erythrocyte sedimentation rate (ESR) [10]. In addition, ADA-positive patients also showed more infusion-related reactions [7, 8]. Therefore, it is clinically important to determine whether or not CT-P13 will increase the positive rate for ADAs after switching.

In this study, we investigated the existence of ADAs before and 52 weeks after switching from IFX to CT-P13. In addition, we compared the baseline characteristics of the ADA-positive and ADA-negative groups.

## Materials and methods

### Patients

This was a prospective observational study. Twenty-nine patients who met the American College of Rheumatology 1987 criteria [11] and treated with intravenous IFX and switched to intravenous CT-P13 at a single center were assessed for their eligibility. All patients had been treated with CT-P13 for 52 weeks except for one patient who had methotrexate-associated pneumonitis. Thus, 28 RA patients were enrolled in this study. The patients’ data were collected from medical records. There was no change in the dose and interval of prior infliximab and CT-P13 in all patients. The study was conducted in accordance with the ethical principles derived from the Declaration of Helsinki and in compliance with the Good Clinical Practice guideline. Inoue Hospital Institutional Review Board approved this study (approval number: 2016-001), and written informed consent for participation was obtained from all patients.

Before and 52 weeks after the start of CT-P13 treatment, the Disease Activity Score based on the 28-joint count (DAS28) and the levels of CRP, matrix metalloproteinase (MMP)-3, and ADA, as well as the ESR were evaluated. Rheumatoid factor (RF) and anti-CCP antibody (ACPA) were investigated at baseline. An enzyme-linked immunosorbent assay kit (Shikari Q-ATI, Matriks Biotek, Turkey) was used for the measurement of the ADA level for both infliximab and CT-P13, and the ADA cut-off value for positivity was set at 10 ng/ml, according to a previous study [12, 13].

### Statistical analyses

The Mann–Whitney U test was used for the comparison of continuous data, and the chi-squared test was used for categorical variables. The paired *t*-test was used to compare characteristics at baseline and 52 weeks after switching. The SPSS Statistics software program, version 25 (IBM Corp., Armonk, NY, USA), was used for the statistical analyses.

## Results

### Baseline characteristics and RA treatment

The baseline characteristics are shown in Table 1. All patients used methotrexate (MTX) and the median dose was 6 (4–8) mg/week. The median duration of prior IFX treatment was 109 (93.5–131.5) months, the median dose of IFX was 6.5 (4.5–8.9) mg/ kg, and the median interval of each IFX administration was 8 (8–9) weeks. The median DAS28 at baseline was 2.9 (2.3–3.2).

**Table 1:** Patient characteristics

	Baseline	After 52-week use of CT-P13	*p* value
Age (years)	69.5 (63–77)		
Gender (male/female), n (%)	7 (25) / 21 (75)		
Body weight (kg)	51.9 (46.6–58.4)		
Disease duration (years)	18 (12.5–27)		
ACPA positive, %	89		
RF positive, %	81		
Steinbrocker class, (1/2/3/4)	7/ 9/ 12/ 0		
Use of MTX, n (%)	28 (100)	28 (100)	
Dose of MTX (mg/week)	6 (4–8)	6 (4–8)	
Use of corticosteroids, n (%)	14 (50)	14 (50)	
Dose of corticosteroids (mg/day)	2.5 (2.5–5)	2.5 (2.5–5)	0.33
Prior IFX treatment duration (months)	109 (93.5–131.5)		
Prior IFX treatment dose (mg/kg)	6.5 (4.5–8.9)		
Prior IFX treatment interval (weeks)	8 (8–9)		
ESR (mm/h)	22.5 (15.3–35.8)	18.5 (8.8–29.8)	0.06
CRP (mg/dL)	0.1 (0.06–0.8)	0.2 (0.07–0.8)	0.93
MMP-3 (ng/ml)	80.2 (39.1–138.7)	66.8 (39.0–106.2)	0.3
DAS28	2.9 (2.3–3.2)	2.7 (1.9–3.1)	0.23

The data are median (interquartile range, Q_1/4_–Q_3/4_) or number (percentage)

*ACPA* anti-CCP antibody, *CRP* C-reactive protein, *DAS28* disease activity score assessing 28 joints, *ESR* erythrocyte sedimentation rate, *IFX* infliximab, *MMP-3* matrix metalloproteinase-3, *MTX* methotrexate, *RF* rheumatoid factor

### Characteristics after 52-week use of CT-P13

The characteristics of patients after 52-week use of CT-P13 are shown in Table 1. The dosages of MTX and corticosteroid were not significantly changed. The median DAS28 at Week 52 was 2.7 (1.9–3.1). There were no serious complications over the 52-week period. The dose and interval of CT-P13 were the same as those of prior IFX treatment.

### Differences in ADA levels at baseline and week 52

**Fig. 1:**
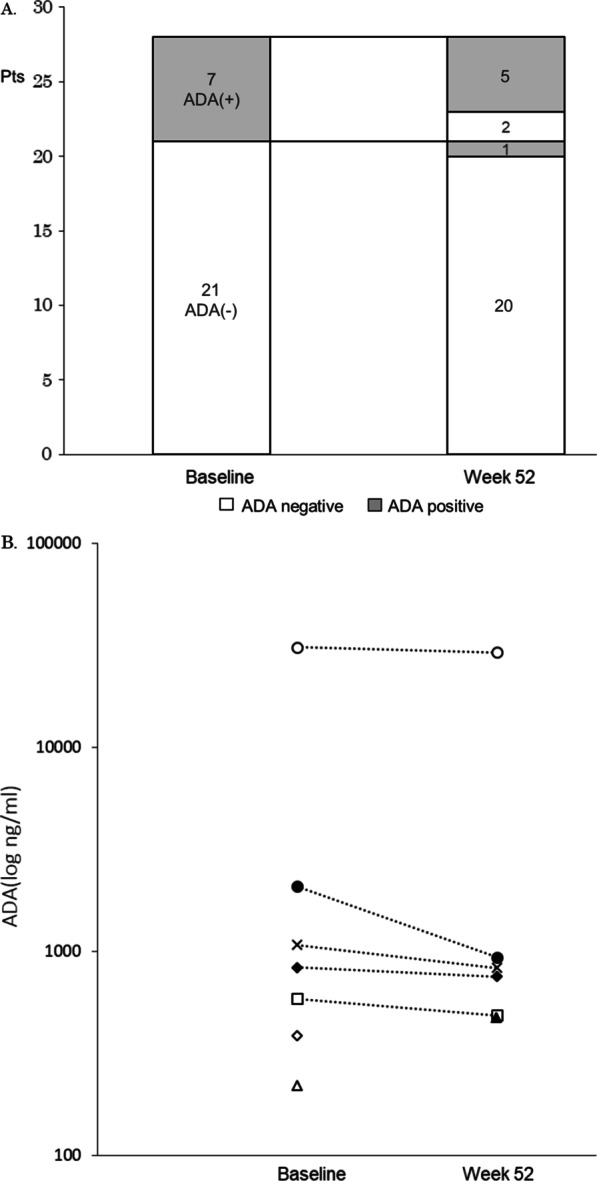
Changes in ADA status at baseline and 52 weeks after switching from IFX to CT-P13 (**A**). Titers of ADA positive patients at baseline and 52 weeks after switching from IFX to CT-P13 (**B**)

At baseline, the ADA-positive rate was 25% (7/28 patients), and 52 weeks after treatment with CT-P13, it was 21.4% (6/28 patients). Two of 7 patients who had ADAs at baseline became negative for ADA, and 1 of 21 patients who had been negative for ADAs at baseline became positive after 52-week use of CT-P13 (Fig. 1A). Switching from IFX to CT-P13 did not significantly increase the positive rate for ADA after 52 weeks. The distribution of ADA titers at baseline and 52 weeks were shown in Fig. 1B. One case with a particularly high titer of ADA was a male patient with a 12-year history of RA who had an ACPA exceeding 400 U/ml; however, his disease activity was suppressed to the point of remission during the observation period.

### ADA-positive patients

Patients were divided into two groups by the existence of ADA at Week 52. The age, sex, RA disease duration, treatment duration of IFX, DAS28, and levels of CRP, ESR, MMP-3, RF, and ACPA at baseline were compared between these two groups. In the ADA-positive group, the value of RF was significantly higher than that in the ADA-negative group (*p* = 0.03) (Table2).

**Table 2:** The comparison of the baseline characteristics between the ADA-positive and ADA-negative groups

	ADA-positive group (n = 6)	ADA-negative group (n = 22)	*p* value
Age (years)	69 (66.3–77.5)	71 (62.5–77)	0.89
Gender (male/female), n	2/4	5/17	0.48
Body weight (kg)	57.5 (44.8–64.7)	51.3 (46.9–55)	0.4
Disease duration (years)	20 (15–32.8)	17.5 (11.8–27)	0.4
Prior IFX treatment duration (months)	102.5 (69.5–127.3)	115.5 (93.8–134.8)	0.45
ESR (mm/h)	27 (12.5–47.5)	20.5 (14.5–33.3)	0.61
CRP (mg/dL)	0.5 (0.1–1.1)	0.1 (0.04–0.5)	0.29
MMP-3 (ng/ml)	91.4 (25.8–211.8)	80.2 (46–118.2)	0.91
DAS28	2.8 (1.9–3.8)	3.0 (2.4–3.2)	0.78
RF (IU/ml)	114.5 (41.3–277.3)	30 (10.5–71)	0.03
ACPA (U/ml)	183.4 (50.2–330)	57.6 (19.9–147.5)	0.18

The data are median (interquartile range, Q_1/4_–Q_3/4_) or number (percentage)

*ACPA* anti-CCP antibody, *CRP* C-reactive protein, *DAS28* disease activity score assessing 28 joints, *ESR* erythrocyte sedimentation rate, *IFX* infliximab, *MMP-3* matrix metalloproteinase-3, *MTX* methotrexate, *RF* rheumatoid factor

## Discussion

In this study, we investigated the incidence of ADAs among RA patients who switched from innovator IFX to biosimilar CT-P13. There was no significant increase in the ADA-positive rate after switching from IFX to CT-P13, and a high titer of RF at baseline was observed in the ADA-positive group (*p* = 0.03).

Previous studies demonstrated that the change from IFX to CT-P13 did not significantly increase ADA incidence [2, 3, 14]. A worldwide randomized, multi-center study demonstrated that the ADA-positive rate after 54-week use of IFX was 48.3% at baseline and 44.8% after 48-week use of CT-P13 [2]. In the Japanese extension study of the clinical trial of CT-P13 (phase I/ II), 33 patients received CT-P13 after 54-week use of IFX. The ADA-positive rate was 48.5% (16/33 patients) at baseline and 21.7% (5/23 patients) after 48 weeks of CT-P13 [3]. Sixteen of 17 patients continued to be negative for ADAs during these 48 weeks, and 5 of 16 patients who were positive for ADAs became negative after 48 weeks in their study. A recent study with a longer observation period also revealed that the ADA-positive rate was 35.6% at baseline and 41.7% after CT-P13 use during the study [14]. In our study, the ADA-positive rates at the time of switching from IFX to CT-P13 and 52 weeks later were 25% and 21.4%, respectively. The dose of IFX and CT-P13 was relatively higher in our study compared to the dose in the previous studies [2, 3], this might influence serum levels of ADA and also the ADA formation.

The DANBIO study, which was based on the Danish nationwide registry, reported that ADA positivity was associated with reduced clinical efficacy and infusion reaction [8]. A low drug concentration [8, 15], long disease duration and smoking [16] have been identified as factors related to ADA positivity in IFX. In addition, a Swedish randomized trial showed that female gender and RF positivity were associated with a low serum IFX concertation and the appearance of ADAs [17]. There was also reported that CT-P13 administered by subcutaneous injection led to the higher serum concentration after 24 weeks and lower ADA positive rate than the CT-P13 by intravenous administration [18].

In our study, the RF values were significantly higher in the ADA-positive group than in the ADA-negative group. RF is an IgM type autoantibody against denatured IgG, and the latest EULAR recommendation has identified it as a poor prognostic factor [19]. A previous randomized double-blind study in Japan revealed that high titers of RF and ACPA were correlated with a low serum IFX level and high TNF level [15]. A high level of RF might thus be associated with a low serum IFX level, leading to the appearance of ADAs. A multi-national, cross-sectional study revealed that IFX-treated patients with ADAs had significantly lower drug concentrations than those without ADAs [10]. The ADA generation was explained by the high TNF level, the higher inflammation or the propensity for antibody production in patients with high RF level.

Several limitations associated with the present study warrant mention. First, the sample size was small, and the observational period was short. Second, the subjects for this study had a long disease duration. Finally, we did not check the drug concentration, although previous reports have shown that ADA positivity was associated with low serum drug concentrations. A further investigation will be needed to clarify the reason for the appearance of ADAs and the treatment effect of CT-P13.

## Conclusion

The ADA-positive rate did not increase after switching from innovator IFX to biosimilar CT-P13. Among the patients with ADA, a high level of RF was observed at baseline.

## Data Availability

The datasets from this study are available from the corresponding author upon request.

## Abbreviations

ACPA: Anti-CCP antibody
ADAs: Anti-drug antibodies
BS: Biosimilar
CRP: C-reactive protein
DAS28: Disease Activity Score based on the 28-joint count
ESR: Erythrocyte sedimentation rate
IFX: Infliximab
MMP: Matrix metalloproteinase
RA: Rheumatoid arthritis
RF: Rheumatoid factor
